# Nonlocal Huygens’ meta-lens for high-quality-factor spin-multiplexing imaging

**DOI:** 10.1038/s41377-024-01728-3

**Published:** 2025-01-30

**Authors:** Jin Yao, Yubin Fan, Yunhui Gao, Rong Lin, Zhihui Wang, Mu Ku Chen, Shumin Xiao, Din Ping Tsai

**Affiliations:** 1 Department of Electrical Engineering, City University of Hong Kong, Kowloon, Hong Kong SAR China; 2 Centre for Biosystems, Neuroscience, and Nanotechnology, City University of Hong Kong, Kowloon, Hong Kong SAR China; 3 State Key Laboratory of Terahertz and Millimeter Waves, City University of Hong Kong, Kowloon, Hong Kong SAR China; 4 State Key Laboratory on Tunable Laser Technology, Ministry of Industry and Information Technology Key Lab of Micro-Nano Optoelectronic Information System, Shenzhen Graduate School, Harbin Institute of Technology, Shenzhen, 518055 China; 5 Pengcheng Laboratory, Shenzhen, China

**Keywords:** Nanophotonics and plasmonics, Metamaterials

## Abstract

Combining bright-field and edge-enhanced imaging affords an effective avenue for extracting complex morphological information from objects, which is particularly beneficial for biological imaging. Multiplexing meta-lenses present promising candidates for achieving this functionality. However, current multiplexing meta-lenses lack spectral modulation, and crosstalk between different wavelengths hampers the imaging quality, especially for biological samples requiring precise wavelength specificity. Here, we experimentally demonstrate the nonlocal Huygens’ meta-lens for high-quality-factor spin-multiplexing imaging. Quasi-bound states in the continuum (q-BICs) are excited to provide a high quality factor of 90 and incident-angle dependence. The generalized Kerker condition, driven by Fano-like interactions between q-BIC and in-plane Mie resonances, breaks the radiation symmetry, resulting in a transmission peak with a geometric phase for polarization-converted light, while unconverted light exhibits a transmission dip without a geometric phase. Enhanced polarization conversion efficiency of 65% is achieved, accompanied by a minimal unconverted value, surpassing the theoretical limit of traditional thin nonlocal metasurfaces. Leveraging these effects, the output polarization-converted state exhibits an efficient wavelength-selective focusing phase profile. The unconverted counterpart serves as an effective spatial frequency filter based on incident-angular dispersion, passing high-frequency edge details. Bright-field imaging and edge detection are thus presented under two output spin states. This work provides a versatile framework for nonlocal metasurfaces, boosting biomedical imaging and sensing applications.

## Abstract (web summary)

We propose a nonlocal Huygens’ meta-lens with a high quality factor, enabling multiplexing functionality for bright-field and edge-enhanced imaging by controlling output spin states.

## Graphical abstract

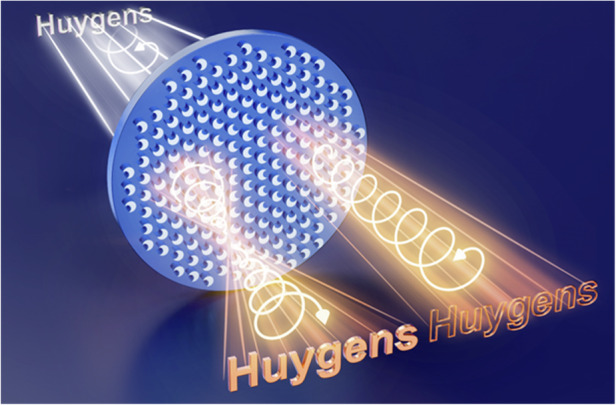

## Introduction

Bright-field and edge-enhanced imaging can provide morphological information of amplitude and phase objects. Combining these two imaging techniques facilitates the detailed visualization of intricate structures, such as biological tissues and cells^1^. Meta-lenses, known for their compact size and flexible electromagnetic manipulation^2^, have been extensively applied in the imaging field, offering unique capabilities such as achromatism^3,4^, tunability^5^, high numerical aperture (NA)^6^, and multimodal perception^7,8^. By incorporating multiplexing or multifunctional focusing and spiral phase profiles, bright-field imaging and edge detection have been integrated into monolithic meta-lenses^9,10^. They can also be electrically switched by liquid crystal cells on the millisecond scale^11^. However, the existing solutions are based on broadband local responses^12,13^, which lack effective modulation of narrowband spectral responses. This limitation can degrade imaging quality due to crosstalk between different wavelengths under broad-spectrum illumination, especially in biomedical samples requiring a specific excitation wavelength.

Nonlocal metasurfaces, which rely on collective resonant responses of multiple meta-atoms, are well-suited for spectral and momentum modulation, particularly in achieving high-quality-factor (high-Q-factor) performance^14,15^. Their preeminent abilities and spatial frequency filtering have led to substantial applications, such as nonlinear generation^16,17^, edge detection^18–21^, and spatial radiation control^22^. By effectively combining the local phase control with nonlocal resonance excitation, nonlocal metasurfaces have been adapted for narrowband wavefront shaping^23^. Phase gradient metasurface with resonant phases, generated through subtle structural perturbations, has been demonstrated to excite high-Q-factor (>2500) guided-mode resonances and steer light to the desired one-dimensional direction^24^, which can be tuned via the electro-optic effect of lithium niobate^25^ or the Kerr effect of silicon^26^. To achieve two-dimensional wavefront shaping, nonlocal metasurfaces with quasi-bound states in the continuum (q-BICs) and geometric phases were proposed to offer both spatial and spectral light control^27^, experimentally realizing a meta-lens with a high Q factor of ~86 and a transmission polarization conversion efficiency of ~4%^28^. Additionally, multispectral responses can be independently controlled by integrating and stacking different metasurfaces, and double-layer designs with Fano resonances further enable the wavefront shapings with higher efficiency or chiral response^29,30^. By incorporating Huygens’ q-BICs, nonlocal meta-lens can simultaneously achieve a high Q factor of 104 and a high efficiency of 55%^31^. Metasurfaces capable of simultaneous spectral and phase modulation are not exclusively reliant on nonlocal mechanisms. They can also be realized through the integration of liquid crystals or other optical components^32^. Nevertheless, the ultimate form is to implement all these functionalities in a single layer of metasurface. Despite these advancements, the performance of nonlocal meta-devices for wavefront shaping - such as Q factor, efficiency, and manipulation dimension - remains constrained by the trade-off between local and nonlocal responses determined by physical mechanism and fabrication precision, and the potential for multifunctionality in these meta-devices has not been explored.

In this work, we experimentally demonstrate a spin-multiplexing high-Q-factor meta-lens for simultaneous bright-field imaging and edge detection in the near-infrared region. The proposed nonlocal Huygens’ meta-lens consists of silicon crescent-shaped integrated-resonant units (IRUs)^33^ on a silica substrate. By introducing asymmetry within the in-plane parametric space, symmetry-protected q-BIC is excited, achieving a high Q factor of 90 and a notable incident-angle dependence. The Fano-like interaction between q-BIC and in-plane Mie-type magnetic dipole resonance (MDR) then results in the generalized Kerker condition, achieving a transmission polarization conversion *T*_RL_ peak with efficiency up to 65% accompanied by a geometric phase robust to the rotation angle of IRUs. The unconverted output polarization *T*_RR_ shows a transmission dip possessing a low value without the geometric phase, surpassing the theoretical limit of traditional nonlocal metasurfaces. These two output spin states are suitably utilized for bright-field imaging based on focusing phase control and edge detection through spatial frequency filtering, respectively, with wavelength-selective properties. This approach ensures minimal interference from other wavelengths, thereby enhancing the accuracy and reliability of the imaging and sensing processes.

## Results

### Principle and design of nonlocal Huygens’ meta-lens

**Fig. 1:**
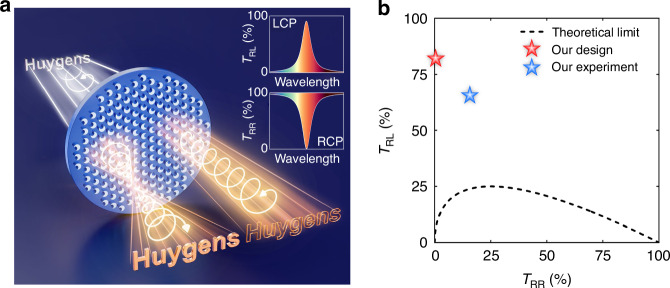
**Spin-multiplexing imaging using nonlocal Huygens’ meta-lens**. **a** Schematic illustration of spin-multiplexing nonlocal Huygens’ meta-lens. Upon illumination with RCP light, the excited nonlocal resonance generates wavelength-selective properties and incident-angle dependence. By geometrically rotating each unit, the output LCP light exhibits a focusing phase profile for bright-field imaging, while the output RCP light without phase modulation is employed for edge detection. Insets are schematic diagrams of spectral responses output LCP and RCP lights required by spin-multiplexing imaging. **b** Functional relationship between transmission efficiencies *T*_RL_ and *T*_RR_ in an ultrathin metasurface according to Eq. (1)

Figure 1a schematically illustrates the proposed nonlocal Huygens’ meta-lens for spin-multiplexing imaging with wavelength-selective properties. With the illumination of right-circularly polarized (RCP) light from the substrate, the transmission polarization-converted left-circularly polarized (LCP) light is utilized for narrowband bright-field imaging, requiring a high-transmission *T*_RL_ peak with a focusing phase profile. In contrast, the unconverted RCP light needs a low-transmission *T*_RR_ dip that approaches zero without the additional phase, exploited for edge-enhanced imaging, which is depicted in the insets of Fig. 1a. *T*_RL(RR)_ is defined as the ratio between the powers of transmission LCP(RCP) and incident RCP lights. However, nonlocal metasurfaces are generally thin with weak out-of-plane asymmetry for the excitation of strong nonlocal resonances, so the relationship between transmission efficiencies *T*_RL_ and *T*_RR_ can be approximately described by^34,35^

1 $${T}_{{\rm{RL}}}={\mathrm{Re}}(\sqrt{{T}_{{\rm{RR}}}})-{T}_{{\rm{RR}}}$$

which is shown in Fig. 1b. It can be seen that the maximum *T*_RL_ is 25% when *T*_RR_ = 25%, and the *T*_RR_ dip to nearly zero can only be acquired as *T*_RL_ is small. Therefore, high *T*_RL_ and low *T*_RR_ cannot be simultaneously achieved in traditional nonlocal metasurface with ultrathin thickness, necessitating our careful design of meta-atoms to overcome this trade-off.

**Fig. 2:**
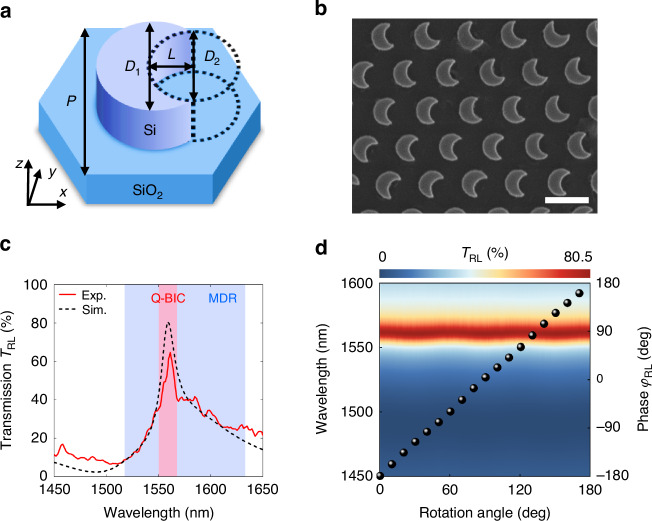
**Design of nonlocal Huygens’ meta-lens**. **a** Schematic diagram of the IRU. **b** SEM image of the fabricated sample. The scale bar is 1000 nm. **c** Experimental and simulated transmission *T*_RL_ spectra. Red and blue areas indicate the bandwidth of q-BIC and MDR, respectively. **d** Dependences of transmission *T*_RL_ (color map) and phase *φ*_RL_ (black spheres) on the rotation angle of IRU. Phases are extracted from the corresponding resonant wavelength

Figure 2a depicts a schematic of the building block, IRU, which comprises the nonlocal Huygens’ meta-lens. The silicon crescent-shaped IRU placed on the silica substrate has a height of 327 nm. It is formed by trimming a cylinder with a diameter of *D*_1_ = 620 nm using another with a diameter of *D*_2_ = 450 nm and an offset of *L* = 220 nm. The hexagonal lattice has an array period of *P* = 1000 nm. A scanning electron microscope (SEM) image of the fabricated sample is shown in Fig. 2b. Figure 2c presents the experimental and simulated transmission *T*_RL_ spectra. Due to asymmetry in the parametric space, nonlocal symmetry-protected q-BIC mode (red area) is excited at a wavelength of 1560 nm. This resonance can be identified by the transmission peak with a high Q factor of 90 (experiment) and 100 (simulation). The local in-plane MDR shows a low-Q-factor transmission peak (blue area) at a resonant wavelength of around 1575 nm. Detailed demonstrations of local and nonlocal effects are discussed in Supplementary Note 5. The interaction between the two resonances results in a Fano-like coupling, achieving the generalized Kerker condition^36^ and a high polarization conversion efficiency of 65% (experiment) and 80.5% (simulation). Experimental and simulated results are in good agreement. Further mode analysis is provided in Supplementary Note 7, based on multipole decompositions^37^. Besides the high Q factor and enhanced efficiency, resonance robustness to the rotation angle that generates the geometric phase is crucial for ensuring a similar response between the IRU and the meta-lens. Figure 2d gives the dependence of transmission *T*_RL_ and phase *φ*_RL_ on the IRU’s rotation angle. The resonance properties are stable, with the resonant wavelength and efficiency remaining constant, and the phase keeps nearly twice the rotation angle. The high Q factor, enhanced efficiency, and stable phase modulation for output LCP light contribute to performant wavelength-selective bright-field imaging.

**Fig. 3:**
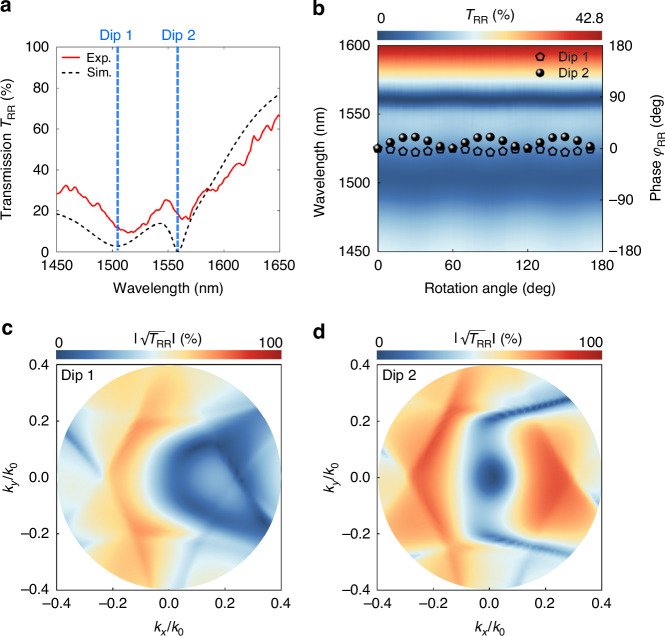
**Optical responses of output RCP light**. **a** Experimental and simulated transmission *T*_RR_ spectra. Blue dashed lines denote wavelengths of two dips. **b** Dependences of transmission *T*_RR_ (color map) and phase *φ*_RR_ (black pentagons and spheres) on the rotation angle of IRU. Phases are extracted from the corresponding resonant wavelength. Simulated transmission coefficients as a function of the normalized in-plane wavevector for Dips 1 (**c**) and 2 (**d**). The collection object has an NA of 0.4, so the in-plane wavevector within this range is considered

For the other output spin state, the generalized Kerker condition allows the transmission *T*_RR_ to reach a low value of nearly zero at the resonant wavelength of q-BIC (Dip 2), as shown in the spectra in Fig. 3a. Associating high *T*_RL_ and low *T*_RR_, the nonlocal Huygens’ meta-lens surpasses the theoretical limit depicted in Fig. 1b. Further discussions on the contribution of generalized Kerker condition are provided in Supplementary Note 6. Due to the interaction between the two resonances, the other transmission *T*_RR_ dip (Dip 1) can also be found. Figure 3b illustrates the dependence of transmission *T*_RR_ and phase *φ*_RR_ on the rotation angle for output RCP light. Similar to Fig. 2d, both dips exhibit stable resonant properties while nearly without phase change. Moreover, nonlocal metasurfaces exhibit interactions between neighboring meta-atoms with extended field distributions, resulting in generally high angular dispersion^38^. Figure 3c and d present the simulated transmission coefficient $\left|\sqrt{{T}_{{\rm{RR}}}}\right|$ as a function of the normalized in-plane wavevector for Dips 1 and 2. To investigate the influence of the metasurface on the electric field intensity, the transmission coefficient $\left|\sqrt{{T}_{{\rm{RR}}}}\right|$ is used here instead of the transmission efficiency *T*_RR_. Under oblique incidence, i.e., at high spatial frequencies, the transmission *T*_RR_ dip undergoes a wavelength shift, causing an increase in transmission at the operational wavelengths. Transmission coefficients of both dips are nearly zero at normal incidence but relatively high for oblique incidence, reaching maximums of 48% and 65%, respectively. This effect enables the potential for spatial frequency filtering, allowing high spatial frequencies carrying object edge information to pass through while blocking central information with low spatial frequencies. Combining these characteristics, the case with the output RCP light is suitable for edge-enhanced imaging. These two spin states have no crosstalk and can be realized simultaneously, which is expected to achieve spin-multiplexing imaging.

### Characterizations of bright-field imaging

**Fig. 4:**
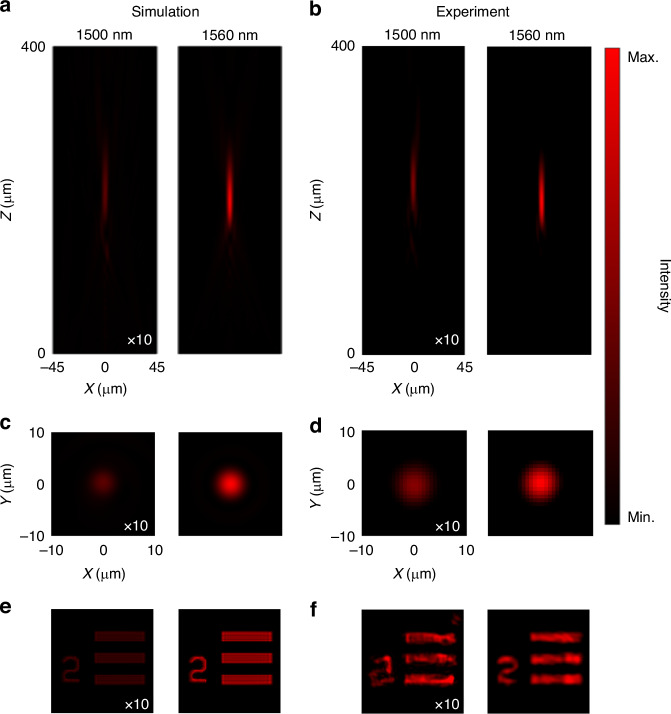
**Wavelength-selective focusing and bright-field imaging of nonlocal Huygens’ meta-lens**. **a**–**f** Simulated and experimental *xz*-plane (**a**, **b**) and *xy*-plane (**c**, **d**) intensity distributions and bright-field imaging (**e**, **f**) at the nonresonant wavelength of 1500 nm and the resonant wavelength of 1560 nm. The *xy* plane is located at the focal plane extracted from *xz*-plane profiles. The intensities at the wavelength of 1500 nm are amplified tenfold for clear observation

IRUs with specific rotation angles are arranged into the nonlocal Huygens’ meta-lens according to the spherical phase profile as follows^39^:

2 $$\varphi =-\frac{2{\rm{\pi }}}{\lambda }\left(\sqrt{{f}^{2}+{r}^{2}}-f\right)$$

where *r* is the radial coordinate, *λ* = 1560 nm is the working wavelength, and *f* = 220 μm is the designed focal length, corresponding to an NA of 0.2 since the diameter of meta-lens is 90 μm. The near-field distribution of the nonlocal Huygens’ meta-lens, shown in Supplementary Fig. S8, demonstrates nearly uniform resonance excitation for each IRU with a different rotation angle. This uniformity is primarily due to rotation robustness, which provides consistent amplitude and target phase, resulting in an effective focusing profile for the meta-lens. Figure 4 gives the focusing profiles in the *xz* and *xy* planes and the bright-field imaging of nonlocal Huygens’ meta-lens. Simulated and experimental results match well with each other. For the resonant wavelength of 1560 nm, the simulated and experimental focal lengths are 220 μm and 211 μm, respectively, consistent with the designed one, as shown in Fig. 4a, b. Their full-width half-maximums in the *xy* plane are 4.1 μm and 4.7 μm (Fig. 4c, d), respectively, indicating a sub-diffraction-limited performance (*λ*/2NA = 3.9 μm). The intensity comparison between resonant and nonresonant wavelengths follows the trend in Fig. 2c, manifesting a significant difference of more than ten folds. Bright-field imaging is performed using the 1951 United States Air Force (USAF) resolution test chart in Fig. 4e, f, revealing effective wavelength-selective properties as well. It should be noted that in the imaging experiment, the imaging quality and the intensity difference between resonant and nonresonant wavelengths are partly diminished due to the influence of the imaging object on the coherence and collimation of the incident light, as well as imperfections in fabrication, which prevent full excitation of the nonlocal resonance. Despite that, with a comparable quality factor and efficiency, the imaging aberration at the resonant wavelength is reduced compared with the reported work^31^, which is more beneficial for practical applications.

### Characterizations of edge-enhanced imaging

**Fig. 5:**
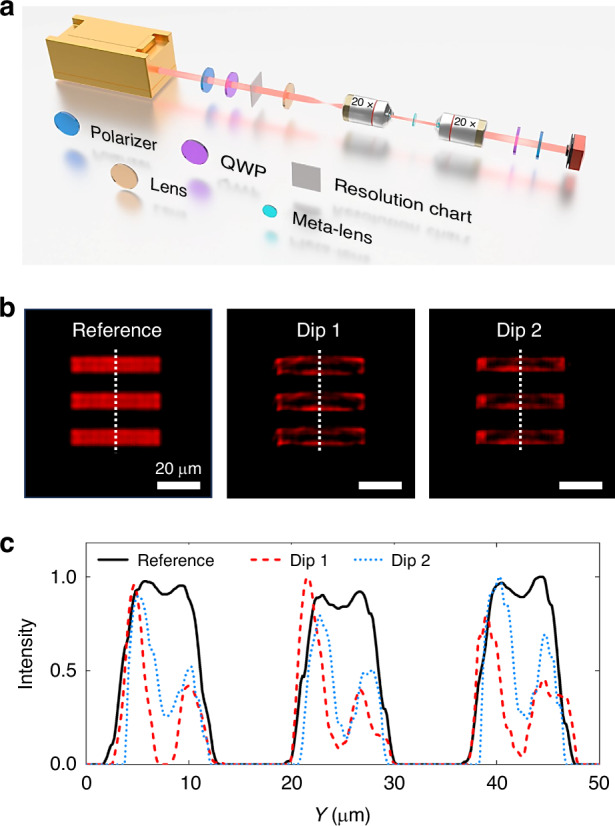
**Edge-enhanced imaging of nonlocal Huygens’ meta-lens**. **a** Experimental setups for edge-enhanced imaging. QWP: quarter-wave plate. **b** Experimental output images of reference, Dip 1, and Dip 2. The reference is the original object imaging without metasurface. Dips 1 and 2 are the scenarios with metasurface filtering, which are extracted from the corresponding wavelengths in Fig. 3a. Scale bars are 20 μm. **c** Intensity distributions along the white dashed lines in (**b)**

Considering the unconverted output RCP light, the experimental setups for edge-enhanced imaging are shown in Fig. 5a. The meta-lens is positioned in the real plane, eliminating the need to place it in the Fourier plane. Figure 5b, c depicts the experimental edge-enhanced imaging and the intensity distribution along the *y* direction. The minimal line size of imaging objects is 7.6 μm. Edges of micrometer-scale images can be observed in both horizontal and vertical directions for both Dips 1 and 2. The intensity distribution along *y*-direction white dashed lines further confirms the edge-enhanced property. It is interesting to point out that Dip 1 has a lower intensity at the image center compared to Dip 2, because Dip 1 is primarily attributed to the MDR with local responses, while Dip 2 is dominated by the nonlocal q-BIC mode, which cannot be fully excited in an imaging scenario. The calculated results are presented in Supplementary Fig. S11 based on the angular spectra in Fig. 3c, d. The deviation between calculation and experiment arises due to several factors: imperfect incident-angle dependence caused by inevitable rotation generating geometric phase, finite size of the meta-lens, and imperfect nonlocal resonance excitation. The calculation method, which relies on spatial filtering and Fourier transform, does not account for these complexities. Theoretical and numerical calculation details of edge detection are given in Supplementary Note 2. Potential approaches include enhancing meta-atom symmetry and reducing the NA of the meta-lens. Associating the bright-field imaging of output LCP light, the functionalities of two output spin states can be achieved without any crosstalk, which maximizes the output light’s utility and facilitates the independent control of versatile imaging applications. Compared with other related works about nonlocal wavefront shaping and multiplexing imaging, our work has a strong performance across four key aspects: Q factor, multiplexity, efficiency, and manipulation dimension, accompanied by the appropriate fabrication requirements. More comparison details are presented in Supplementary Note 11. The proposed meta-lens holds the potential for compatibility with mass production processes, allowing for expansion into various meta-devices^40–42^ and their designs^43–45^, as well as to different wavelength ranges including ultraviolet^46,47^, visible^48^, and infrared regions^49^.

## Discussion

To conclude, we propose the nonlocal Huygens’ meta-lens employed for wavelength-selective bright-field and edge-enhanced imaging by selecting the output spin state. The carefully designed IRU serves to not only excite a high-Q-factor (90) q-BIC mode with incident-angle dependence but also generate the generalized Kerker condition through the interaction between q-BIC mode and MDR, resulting in an experimental high-efficiency (65%) peak and two low-transmission dips for the two output spin states, respectively. In addition, the design ensures geometric rotation robustness, providing stable phase modulation and minimal phase change for two spin states, respectively. Benefiting from these effects, the wavelength-selective focusing and bright-field imaging are demonstrated to have an efficiency enhanced by at least ten folds at resonant wavelength compared to the nonresonant one. The other output spin state is utilized for edge-enhanced imaging, capable of resolving micrometer-scale objects. Compared to previous work^31^, this work further demonstrates the subtle interplay between the Kerker effect and multiplexing functionality. Future development might include exploring other possible IRUs and expanding the work to different wavelength ranges, such as terahertz and visible light. More details are given in Supplementary Note 12. Further enhancements of the nonlocal Huygens’ meta-lens could involve optimization algorithms and artificial intelligence techniques to manipulate nonlocal effects and minimize their impact on imaging^50^. More nonlocal effects, such as surface plasmon polaritons and surface lattice resonances, could be explored to strengthen the functionality^38^. Introducing high-order Mie resonances presents a potential approach to surpass the nonlocal limit^51^. The proposed nonlocal Huygens’ meta-lens paves the way for performant high-Q-factor wavefront shaping and image processing. Spin-multiplexing imaging with wavelength-selective properties holds promise for practical applications in complex biomedical imaging, sensing, and microscopy.

## Methods

### Simulation

The electromagnetic responses of IRUs and meta-lenses are numerically simulated using COMSOL Multiphysics, leveraging the finite element method. To truncate the open space, perfectly matched layers (PMLs) are implemented at the top and bottom of the structure. Periodic boundary conditions are applied in the *x* and *y* directions to simulate the periodic IRU configuration. For the near-field simulation of single IRU and meta-lenses, these boundary conditions are replaced with PMLs. Far-field profiles are derived by combining near-field simulation results with scalar diffraction theory for light propagation. The refractive index of silicon is detailed in Supplementary Fig. S1, while the refractive index of the silica substrate is 1.45.

### Fabrication

The detailed steps involved in the fabrication of the silicon nonlocal Huygens’ meta-lens are illustrated in Supplementary Fig. S3. Initially, a 327-nm-thick α-Si film is deposited onto a SiO_2_ substrate at a rate of 0.5 Å s^−1^. This is followed by the deposition of a 22-nm-thick Cr layer, acting as a hard mask, at the same rate using an electron beam evaporator. Subsequently, an 80 nm PMMA film is spin-coated onto the Cr layer and then baked at 180 °C for one hour. The PMMA photoresist is then patterned by exposure to an electron beam (Raith E-line, 30 kV) and developed in a MIBK/IPA solution for 30 seconds at 0 °C to create PMMA nanostructures. Following the development of the photoresist, inductively coupled plasma (ICP) etching (Oxford ICP180) is employed to etch both the Cr and Si layers sequentially. Finally, the residual Cr film is removed by immersing the sample in a chromium etchant for 10 minutes.

### Characterization

The specific configurations for optical measurement are illustrated in Supplementary Fig. S4. A supercontinuum laser (NKT, FIU-6) serves as the source of broadband coherent light. To achieve single-wavelength illumination for light-field focusing and imaging, an acousto-optic tunable filter is incorporated. Circular polarization generation is achieved through a combination of a linear polarizer and a quarter-wave plate. A lens, together with an object lens (Mitutoyo, 20× magnification, NA = 0.4), produces collimated incident light. The influence of collimation and coherence is discussed in detail in Supplementary Note 9. For the collection of transmitted light, an identical object lens (Mitutoyo, 20× magnification, NA = 0.4) is utilized. Following transmission through the quarter-wave plate and linear polarizer, the spectrometer or camera can analyze the respective LCP and RCP components.

## Supplementary information

Supplementary Information

## Data Availability

Data underlying the results are available from the corresponding authors upon request.
