# Supplementary material for: Nonlocal Huygens’ meta-lens for high-quality-factor spin-multiplexing imaging
Source: Light Sci Appl. 2025 Jan 30;14:65. doi: 10.1038/s41377-024-01728-3 (PMC11782524; doi:10.1038/s41377-024-01728-3)
Supplement: Supplementary file 1 — Supplementary Information [file 41377_2024_1728_MOESM1_ESM.docx]

**Supplementary Information**

Nonlocal Huygens’ meta-lens for high-quality-factor spin-multiplexing imaging

Jin Yao,^1,†^ Yubin Fan,^1,†^ Yunhui Gao,^1^ Rong Lin,^1^ Zhihui Wang,^1^ Mu Ku Chen,^1,2,3^ Shumin Xiao,^4,*^ and Din Ping Tsai^1,2,3,*^

*1 Department of Electrical Engineering, City University of Hong Kong, Kowloon, Hong Kong SAR, China*

*2 Centre for Biosystems, Neuroscience, and Nanotechnology, City University of Hong Kong, Kowloon, Hong Kong SAR, China*

*3 State Key Laboratory of Terahertz and Millimeter Waves, City University of Hong Kong, Kowloon, Hong Kong SAR, China*

*4 State Key Laboratory on Tunable Laser Technology, Ministry of Industry and Information Technology Key Lab of Micro-Nano Optoelectronic Information System, Shenzhen Graduate School, Harbin Institute of Technology, Shenzhen 518055, China*

**^*^**Corresponding Authors**,** E-mails: shumin.xiao@hit.edu.cn; dptsai@cityu.edu.hk

^†^These authors contributed equally to this work.

Supplementary Note 1: Refractive index of silicon

Supplementary Note 2: Theoretical and numerical calculations of edge detection

Supplementary Note 3: Fabrication process

Supplementary Note 4: Measurement setups

Supplementary Note 5: Demonstration of the nonlocal effect

Supplementary Note 6: Demonstration of the contribution from generalized Kerker condition

Supplementary Note 7: Multipole decompositions of the proposed IRU

Supplementary Note 8: Near-field distribution

Supplementary Note 9: Influence of collimation and coherence

Supplementary Note 10: Calculated edge-enhanced imaging

Supplementary Note 11: Performance comparison

Supplementary Note 12: Meta-atom design in the visible

# Supplementary Note 1: Refractive index of silicon


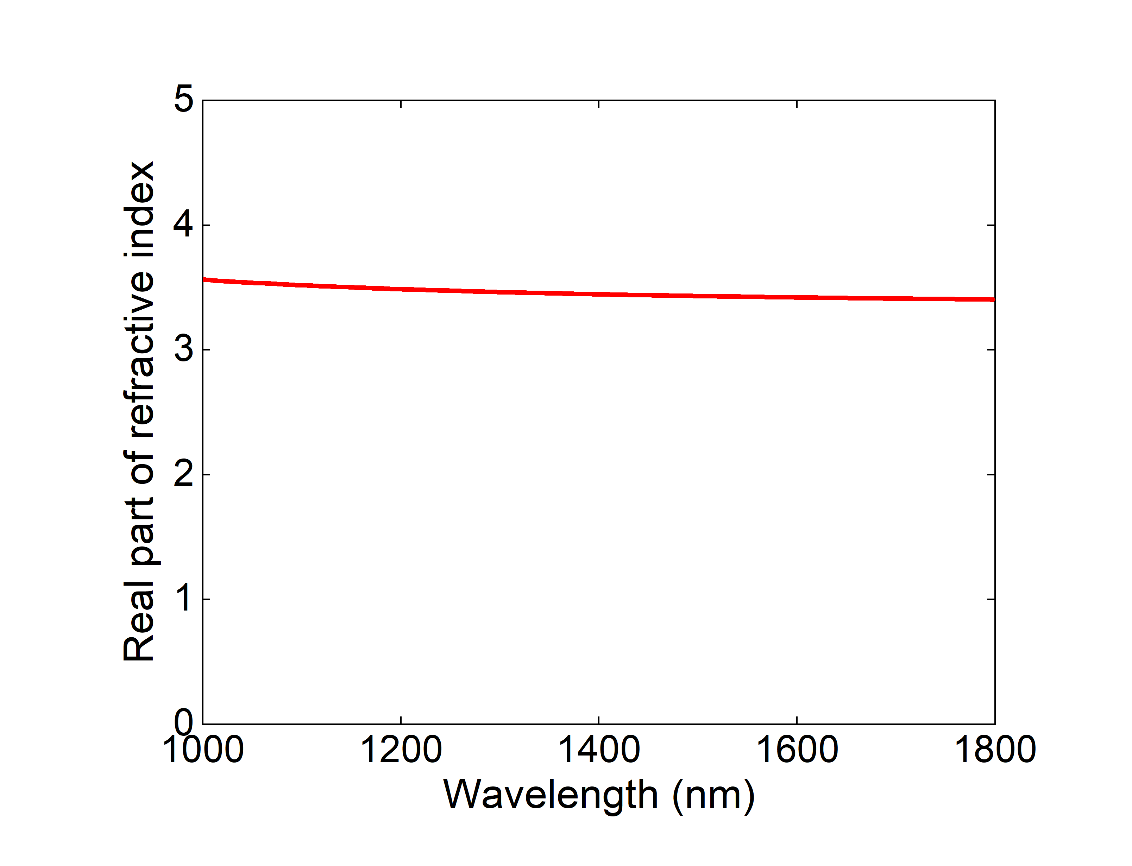


**Fig. S1:** The real part of the refractive index of silicon film obtained through the optical measurement using an ellipsometer. The imaginary part is zero for the target wavelength.

# Supplementary Note 2: Theoretical and numerical calculations of edge detection


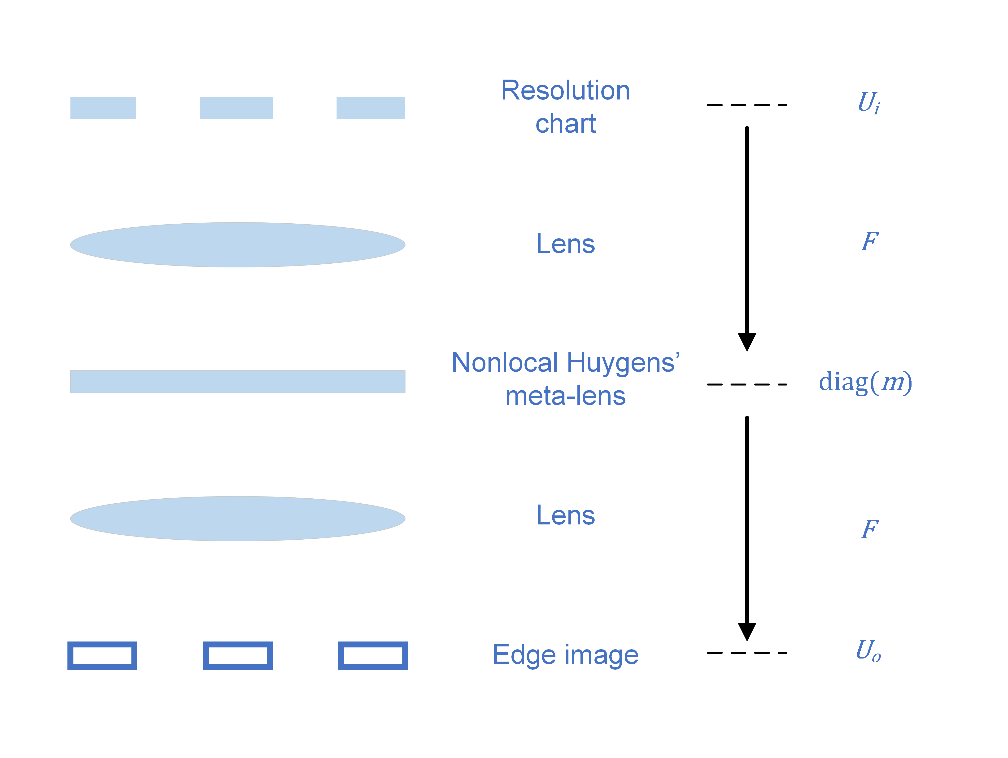


**Fig. S2: Illustration of the edge detection model.** Diagram of the setup mathematical operations.

In this section, we delineate the specific mathematical procedures essential for computing the image processing effects induced by nonlocal Huygens’ meta-lens. We elucidate the methodology employed to generate the results depicted in Fig. 5 of the main manuscript. The derivation adheres closely to the framework outlined in Supplementary Ref. 1.

We start by considering (Fig. S2) that a resolution target is characterized at the plane *z* = 0 by a spatially varying intensity profile $I_{in}\left( x,y \right)=\left| \mathbf{E}_{in}\left( x,y \right) \right|^{2}$, where $\mathbf{E}_{in}\left( x,y \right)=E_{in}\left( x,y \right)\hat{e}$ represents an electric field with polarization state $\hat{e}$. To provide a concrete example, the image can be conceptualized as being produced by a plane wave with polarization $\hat{e}$ passing through an aperture, although the ensuing calculations are applicable regardless of the image generation method. The Fourier transform of the input image is delineated as $\mathcal{F\{}E_{i}\left( x,y \right)\}=\int E_{i}\left( x,y \right)e^{-i\left( k_{x}x+k_{y}y \right)}dxdy$, where $\left[ k_{x},k_{y} \right]$ denote the in-plane wave vector components. Based on the thin lens approximation, the front and back focal planes of a lens are related by a Fourier transform. We define the Fourier transform as $U_{o}\mathcal{=F\{}U_{i}\mathcal{\}=F\cdot}U_{i}$, where $U_{i}$ and $U_{o}$ denote the input and output complex field and $\mathcal{F}$ denote the Fourier transform. The image can be decomposed into an assemblage of plane waves, each propagating in a direction defined by the in-plane wave vector components.

We examine the scenario of edge detection by the nonlocal Huygens’ meta-lens, for which the transfer functions are defined in Fig. 3c and 3d. The nonlocal Huygens’ meta-lens introduces a momentum modulation to the incident wavefield $U_{o}=M\cdot U_{i}$, where $M$ represents the transmission function of the nonlocal Huygens’ meta-lens.

Finally, the edge detection model is derived by sequentially applying all the operators as follows:

$$\begin{aligned} U_{o}\mathcal{=F\cdot}{M\mathcal{\cdot F\cdot}U}_{i}\#\left( 1 \right) \end{aligned}$$

# Supplementary Note 3: Fabrication process


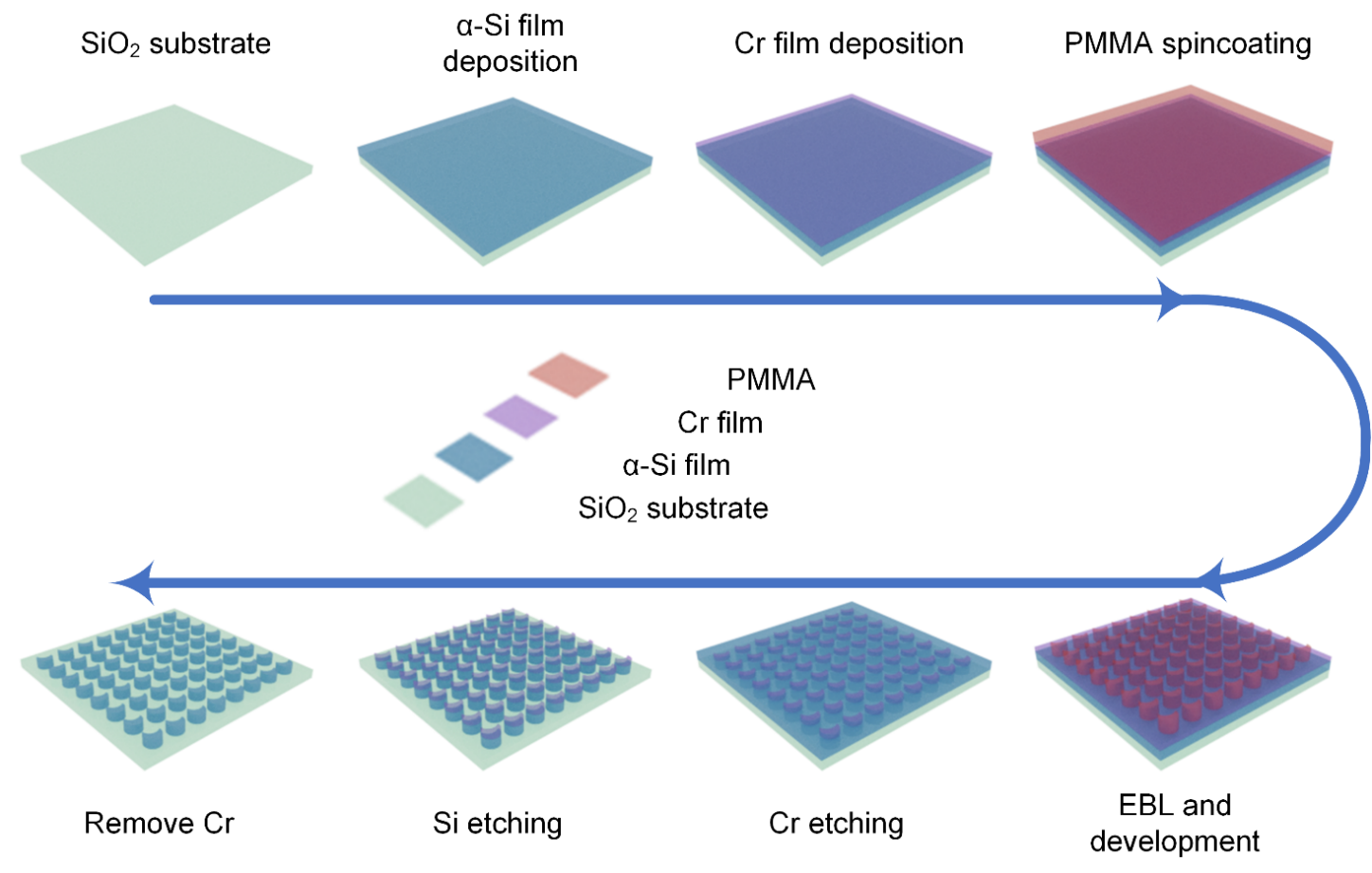


**Fig. S3:** The fabrication process of nonlocal Huygens’ meta-lens.

The detailed fabrication process of the silicon meta-lens is outlined in Fig. S3. Initially, a 327 nm α-Si film is deposited onto a SiO_2_ substrate at a deposition rate of 0.5 Å s^-1^. Subsequently, a 22 nm Cr film is applied as a hard mask using an electron beam evaporator, also at a deposition rate of 0.5 Å s^-1^. Following this, an 80 nm PMMA film is spin-coated onto the substrate and baked at 180 °C for one hour. The PMMA resist is then exposed to an electron beam (Raith E-line, 30 kV) and developed in a MIBK/IPA solution at 0 °C for 30 seconds to create the desired PMMA nanostructures. After the development of the resist, inductively coupled plasma (Oxford ICP180) is employed to etch the Cr and Si layers sequentially. In the final step, the residual Cr film is removed by immersing the sample in a chromium etchant for 10 minutes.

# Supplementary Note 4: Measurement setups


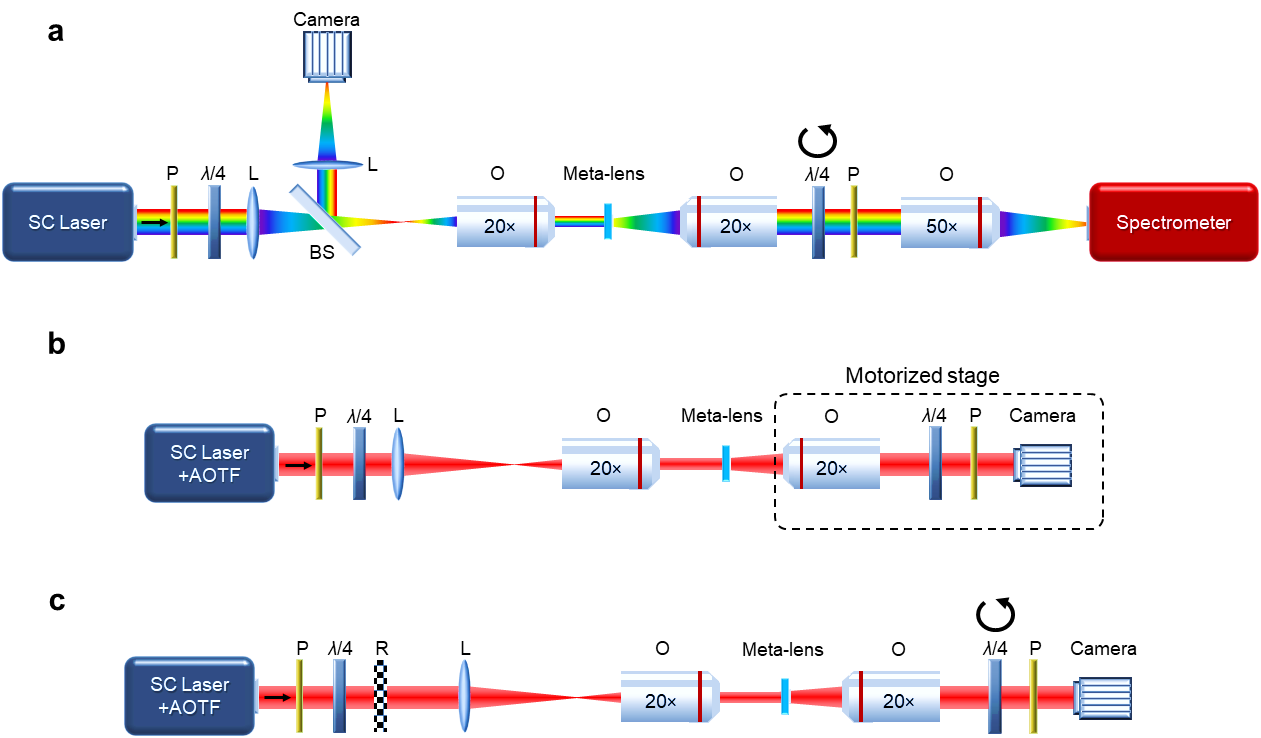


**Fig. S4:** **a**,**b**,**c**, Optical measurement setups for transmission spectrum (**a**), light intensity distribution (**b**), and bright-field and edge-enhanced imaging (**c**). SC Laser: Supercontinuum laser, P: Linear polarizer, L: Lens, BS: Beam splitter, AOTF: Acousto-optic tunable filter, O: Objective, *λ*/4: Quarter-wave plate, R: Resolution chart.

Supercontinuum laser is used to provide wideband coherent light. To generate circular polarization, a linear polarizer and a quarter-wave plate are utilized. The incident light is collimated using a lens in conjunction with an object possessing 20× magnification and a numerical aperture (NA) of 0.4. This same object is also employed to collect the transmitted light. After the light passes through the quarter-wave plate and the linear polarizer, the intensities of the left circularly polarized (LCP) and right circularly polarized (RCP) components can be measured by rotating the quarter-wave plate (shown in optical measurement setups in Fig. S4). These measurements are conducted using either a spectrometer or a camera, enabling detailed analysis of the light's polarization characteristics.

# Supplementary Note 5: Demonstration of the nonlocal effect


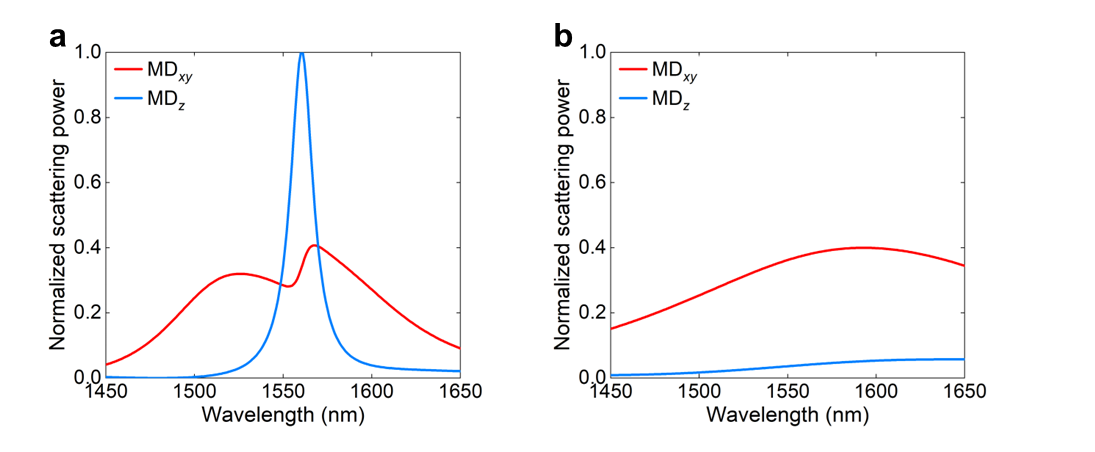


**Fig. S5: Demonstration of the nonlocal effect** **in meta-lens.** **a**,**b**, In-plane (MD*_xy_*) and out-of-plane (MD*_z_*) magnetic dipole moments of the periodic IRU (**a**) and the single IRU (**b**).

Figures 5a and 5b give the in-plane and out-of-plane magnetic dipole moments of the periodic integrated-resonant unit (IRU) and the single IRU, respectively. For periodic IRU, low-quality-factor (low-Q-factor) in-plane magnetic dipole resonance (MDR) and high-Q-factor quasi-bound state in the continuum (q-BIC) mode can both be observed. Without the nonlocal interaction with neighboring IRUs, a single IRU can only excite the low-Q-factor MDR but the high-Q-factor q-BIC mode cannot be effectively excited, which confirms the presence and significance of the nonlocal effect.

# Supplementary Note 6: Demonstration of the contribution from generalized Kerker condition


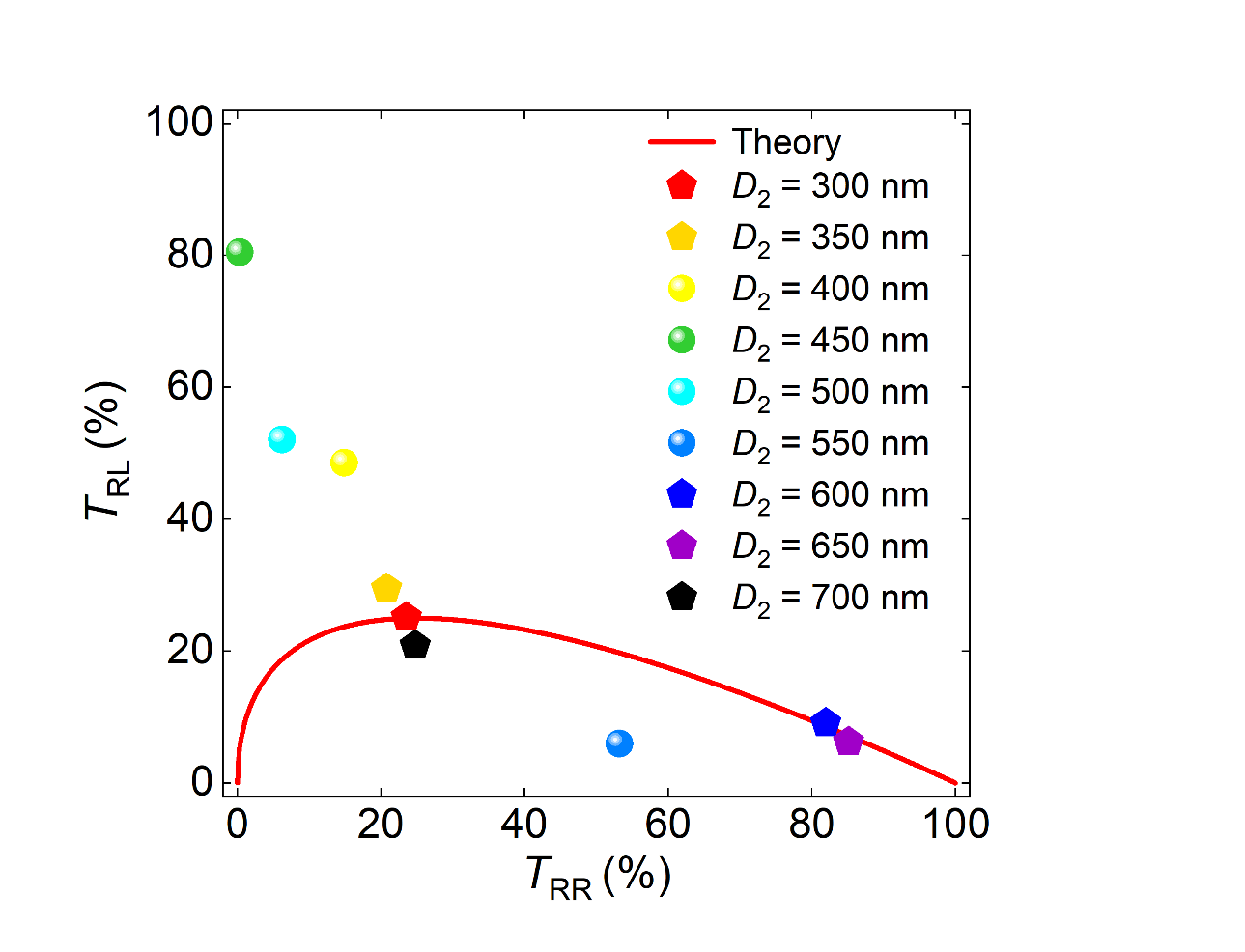


**Fig. S6:** Simulated transmission *T*_RR_ and *T*_RL_ of q-BICs at resonant wavelengths with different diameters *D*_2_. Spheres and pentagons denote the cases within and outside the range of the generalized Kerker condition, respectively. The theoretical curve is identical to that in Fig. 1b in main text.

Breaking the out-of-plane symmetry can overcome the efficiency limit of 25%, which generally needs the height of the meta-atom in local dielectric meta-lenses to reach the wavelength level. This type of meta-atom can generate the waveguide-like mode and the polarization conversion efficiency is thus not limited.

For the IRU designed in our work, the height is only around a fifth of the wavelength, and the refractive index of the substrate is 1.45, which does not differ much from that of the air. Moreover, the high-Q-factor q-BIC mode is dominated by the vertical magnetic dipole moments, which are contributed by the in-plane current. Therefore, the out-of-plane symmetry break is weak. To demonstrate this point, the simulated transmission *T*_RR_ and *T*_RL_ of q-BICs at resonant wavelengths for different diameters *D*_2_ are shown in Fig. S6. When *D*_2_ > 550 nm or *D*_2_ < 400 nm, without the interaction with MDR, only the single mode of q-BIC is excited, resulting in the relationship between *T*_RR_ and *T*_RL_ close to the theoretical line. Therefore, a high *T*_RL_ and a low *T*_RR_ cannot be simultaneously achieved. When the diameter *D*_2_ is between around 400 nm and 550 nm, MDR couples with the q-BIC mode, generating the generalized Kerker condition. The simulated *T*_RR_ and *T*_RL_ show large deviations from the theoretical values. The maximum *T*_RL_ = 81.5% with *D*_2_ = 450 nm is acquired accompanied by the minimum *T*_RR_ close to zero. This phenomenon confirms that the designed unit has intrinsically weak out-of-plane asymmetry and demonstrates the significance of the generalized Kerker condition.

# Supplementary Note 7: Multipole decompositions of the proposed IRU


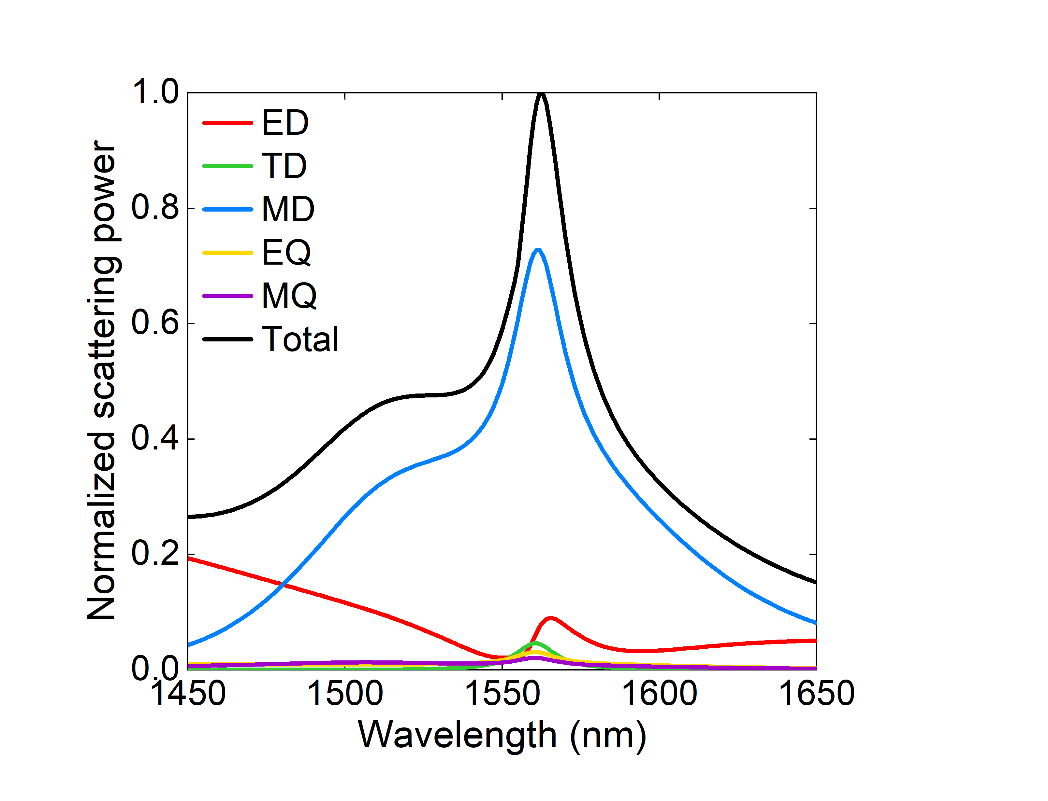


**Fig. S7:** Multipole decompositions of the proposed IRU. ED: electric dipole, TD: toroidal dipole, MD: magnetic dipole, EQ: electric quadrupole, MQ: magnetic quadrupole.

The vertical MDR, i.e. the BIC mode, is originally a dark mode for normal incidence. By breaking the symmetry in the parameter space, the q-BIC mode is effectively induced by asymmetric horizontal dipole moments. The vertical magnetic dipole moments cannot generate vertical radiation, but other horizontal multipole moments induced by the q-BIC mode can influence the normal transmission, such as electric dipole, toroidal dipole, etc., which can be found by the peak in the scattering spectra of multipoles in Fig. S7. These multipoles interfere with the horizontal magnetic dipole induced by the in-plane MDR, generating the generalized Kerker condition to break the radiation symmetry and improve the transmission efficiency.

# Supplementary Note 8: Near-field distribution


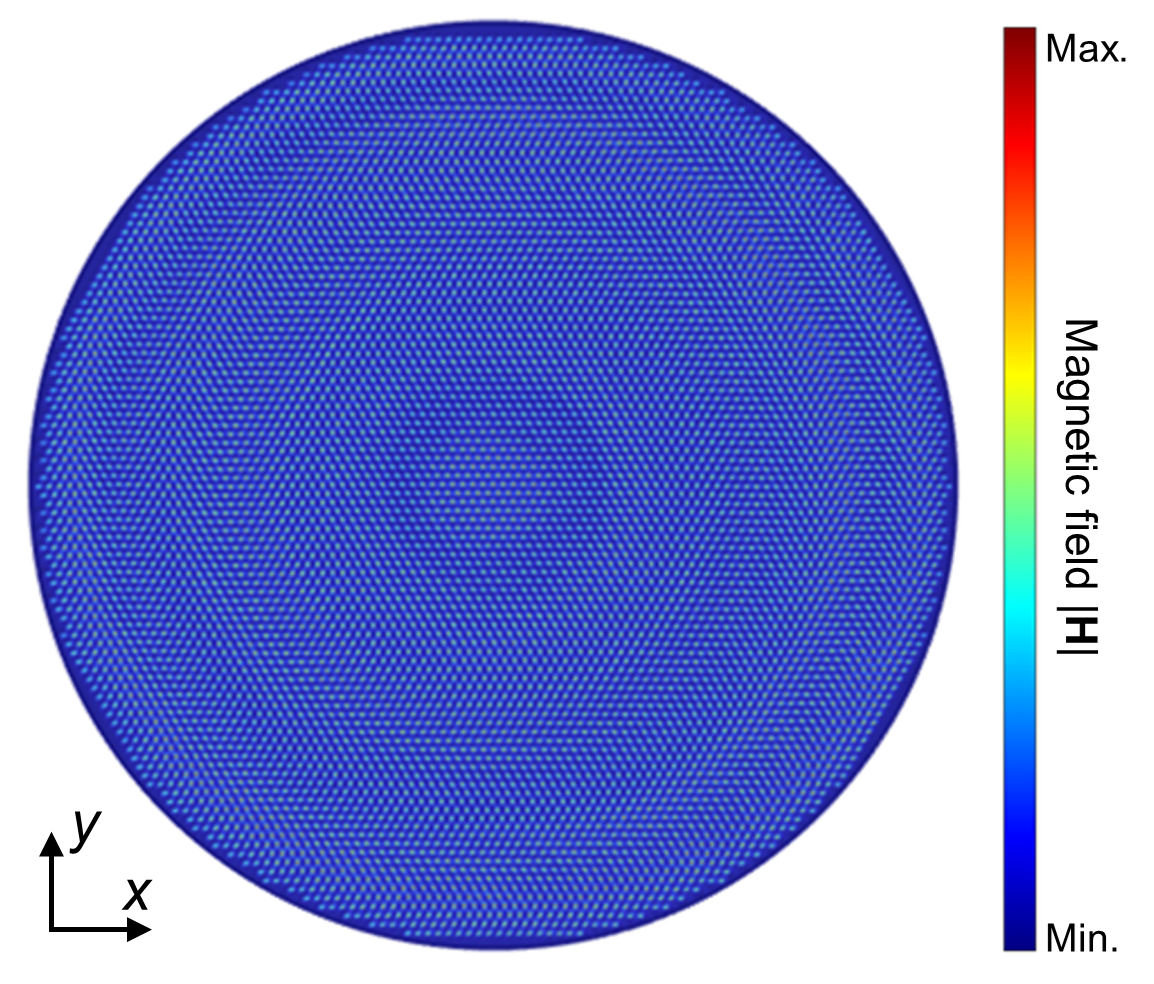


**Fig. S8:** Magnetic field distribution |**H**| of the nonlocal Huygens’ meta-lens. The *xy* cut plane is located at the center of the meta-lens.

The near-field distribution of the nonlocal Huygens’ meta-lens is shown in Fig. S8. Magnetic field |**H**| is chosen because two resonances of q-BIC mode and MDR can both generate the magnetic hotspot inside the IRU. Each IRU with a different rotation angle at the target position possesses approximately uniform resonance excitation strength. This uniformity is primarily due to rotation robustness, which provides consistent amplitude and target phase, resulting in an effective focusing profile for the meta-lens in Fig. 4.

# Supplementary Note 9: Influence of collimation and coherence


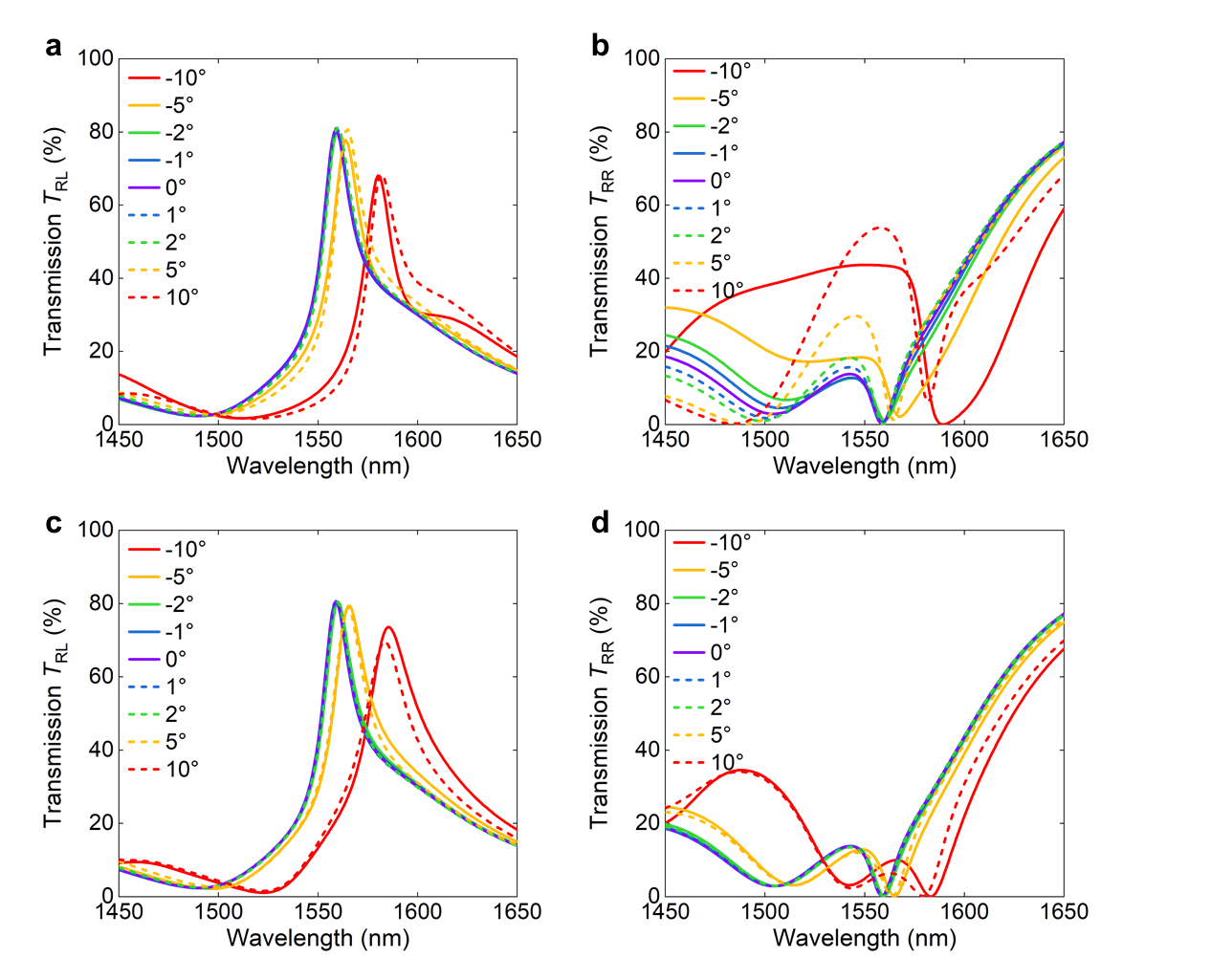


**Fig. S9: Transmission spectra at different incident angles. a**,**b**, *T*_RL_ (**a**) and *T*_RR_ (**b**) in *xoz* plane. **c**,**d**, *T*_RL_ (**c**) and *T*_RR_ (**d**) in *yoz* plane.

The nonlocal response of the proposed meta-lens introduces incident-angle dependence, which is crucial for achieving edge-enhanced imaging. However, this angular dispersion also presents challenges for practical measurement. Figure S9 shows the transmission spectra at different incident angles, revealing angular dispersion that reduces the quality factor under focused illumination. Precise collimation with an oblique angle smaller than 5° is therefore critical for ensuring effective nonlocal resonance excitation.


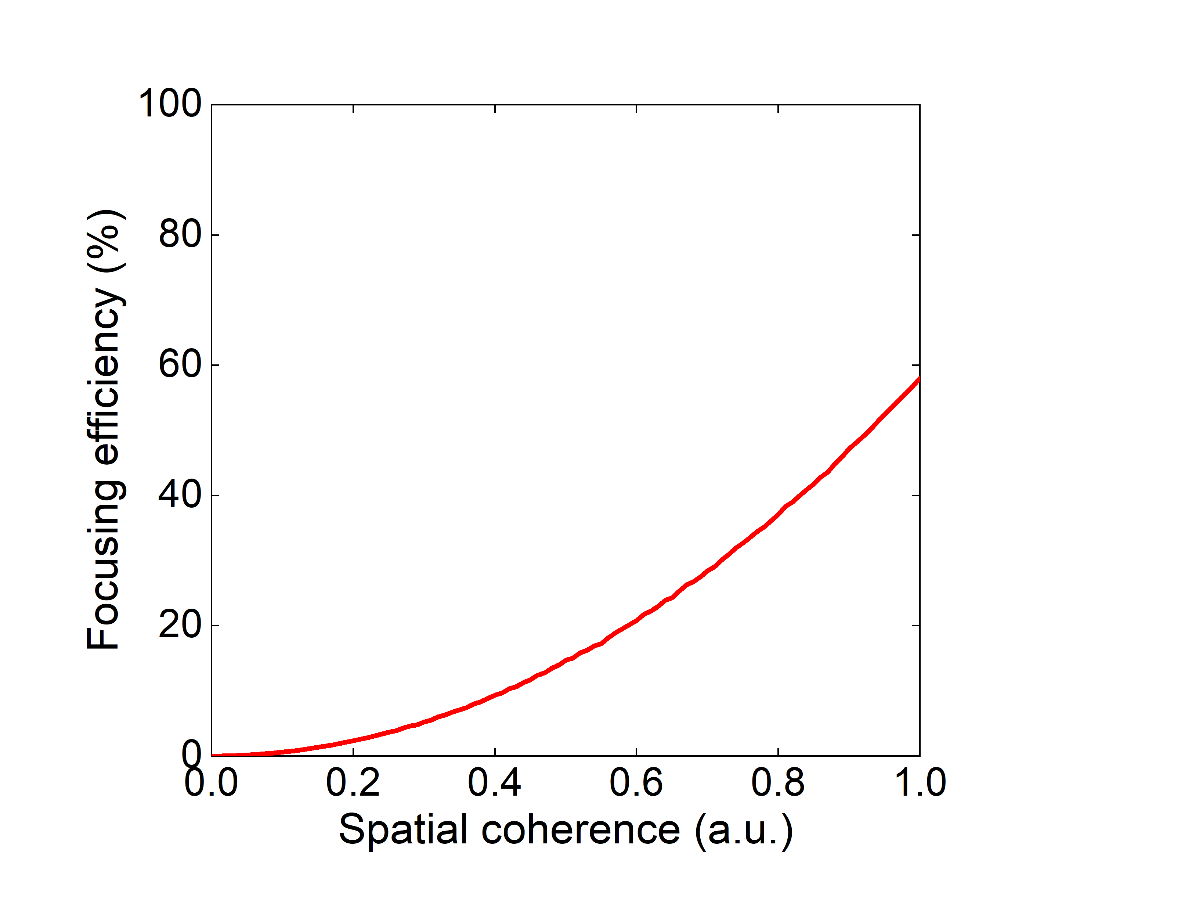


**Fig. S10: Focusing efficiency as a function of spatial coherence of incident light**. Focusing efficiency is defined as the ratio of the total intensity within three times the full width at half maximum of the focal spot to the incident light intensity. Spatial coherence refers to the fraction of the incident wavefront that maintains an undisturbed phase.

Since the nonlocal effect relies on the resonant coupling between adjacent meta-atoms, the coherence of the incident light is key to enabling effective resonance responses and interactions in both time and spatial dimensions, which cannot be overlooked in practical operations. For spatial coherence, the relationship between the focusing efficiency of the meta-lens and the spatial coherence of the incident light is illustrated in Fig. S10. To simulate spatial incoherence, random phase disturbances are introduced to the incident light. As coherence decreases, the focusing efficiency diminishes, highlighting the importance of spatial coherence. Regarding temporal coherence, the resonant bandwidth of the meta-lens defines the limit. A narrower laser linewidth results in better temporal coherence. If the laser linewidth is narrower than the meta-lens's resonant bandwidth, coherence remains unaffected. However, when the linewidth exceeds the resonant bandwidth, a portion of the light is not effectively utilized. Furthermore, the importance of temporal coherence depends on the specific application. When the meta-lens is employed as a light source, temporal coherence becomes less critical.

# Supplementary Note 10: Calculated edge-enhanced imaging


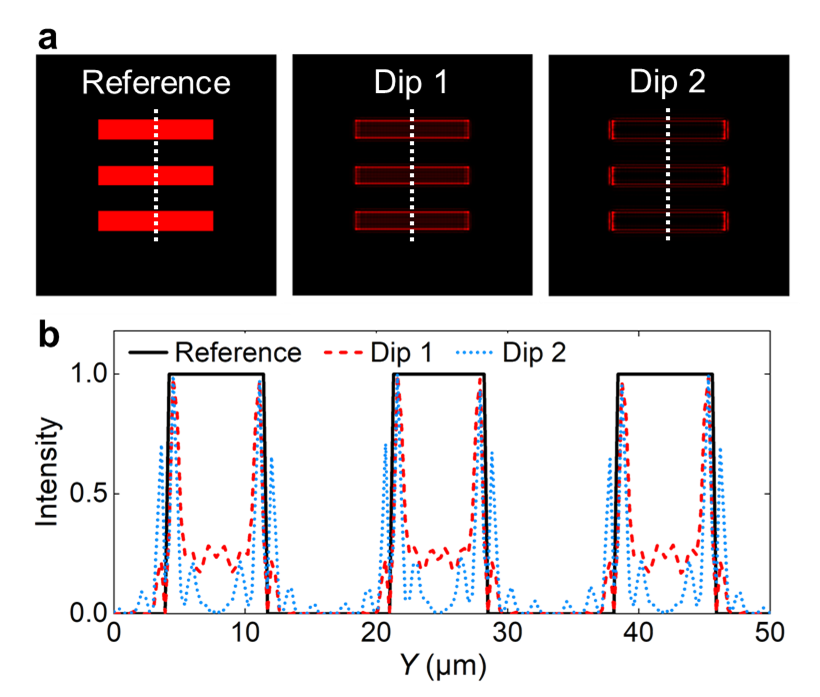


**Fig. S11: Calculated edge-enhanced imaging of nonlocal Huygens’ meta-lens. a**, Output images of reference, Dip 1, and Dip 2. The reference is the original object imaging without metasurface. Dips 1 and 2 are the scenarios with metasurface filtering, which are extracted from the corresponding wavelengths in Fig. 3a. **b**, Intensity distributions along the white dashed lines in Fig. S11a.

# Supplementary Note 11: Performance comparison

**Table S1.** Performance comparison between meta-devices for nonlocal wavefront shaping and multiplexing imaging.

| References | Q factor | Multiplexity | Efficiency | Wavefront shaping dimension |
| --- | --- | --- | --- | --- |
| This work | 90 | √ | 65% | 2D |
| Ref. 2 | ~86 | × | ~4% | 2D |
| Ref. 3 | >2500 | × | ~20% | 1D |
| Ref. 4 | ~380 | × | 59% | 1D |
| Ref. 5 | Broadband | √ | 50% | 2D |
| Ref. 6 | Broadband | √ | No data | 2D |
| Ref. 7 | Broadband | √ | <32.3% | 2D |

For the performance comparison with related works, it is important to highlight the advantages and differences of our work as follows:

1. Advantages

Compared with existing meta-devices for nonlocal wavefront shaping, our work simultaneously ensures four aspects of the Q factor, multiplexity, efficiency, and wavefront shaping dimension. With a comparable Q factor, our efficiency is more than one order of magnitude higher than Ref. 2 based on the q-BIC metasurface. By employing the geometric phase, our work can achieve two-dimensional wavefront shaping, while Refs. 3 and 4 based on meta-gratings with guided-mode resonances only demonstrate one-dimensional control, and our fabrication process does not require extreme precision of up to a few nanometers. Moreover, for the first time, we propose the multiplexing functionality in nonlocal meta-lenses. Compared with previous meta-devices for multiplexing imaging, we realize a high Q factor of 90 instead of broadband responses in Refs. 5-7. This filtering property reduces the crosstalk between different wavelengths, which can enhance the imaging quality in biomedical samples requiring a specific excitation wavelength.

1. Differences

The physical mechanisms of our work and this reported work are intrinsically distinct. Our work employs q-BIC mode to acquire a high Q factor with incident-angle dependence. The generalized Kerker effect is carefully designed to offer a high-efficiency peak and two low-transmission dips for two output spin states, respectively, which break the limit in existing nonlocal metasurfaces. The rotation robustness for the geometric phase is optimized as well. These two output spin states are suitably utilized for bright-field imaging based on focusing phase control and edge detection through spatial frequency filtering, respectively.

Combining four aspects of the Q factor, multiplexity, efficiency, and manipulation dimension, as well as the fabrication requirements, our work has a strong performance compared with other works.

# Supplementary Note 12: Meta-atom design in the visible


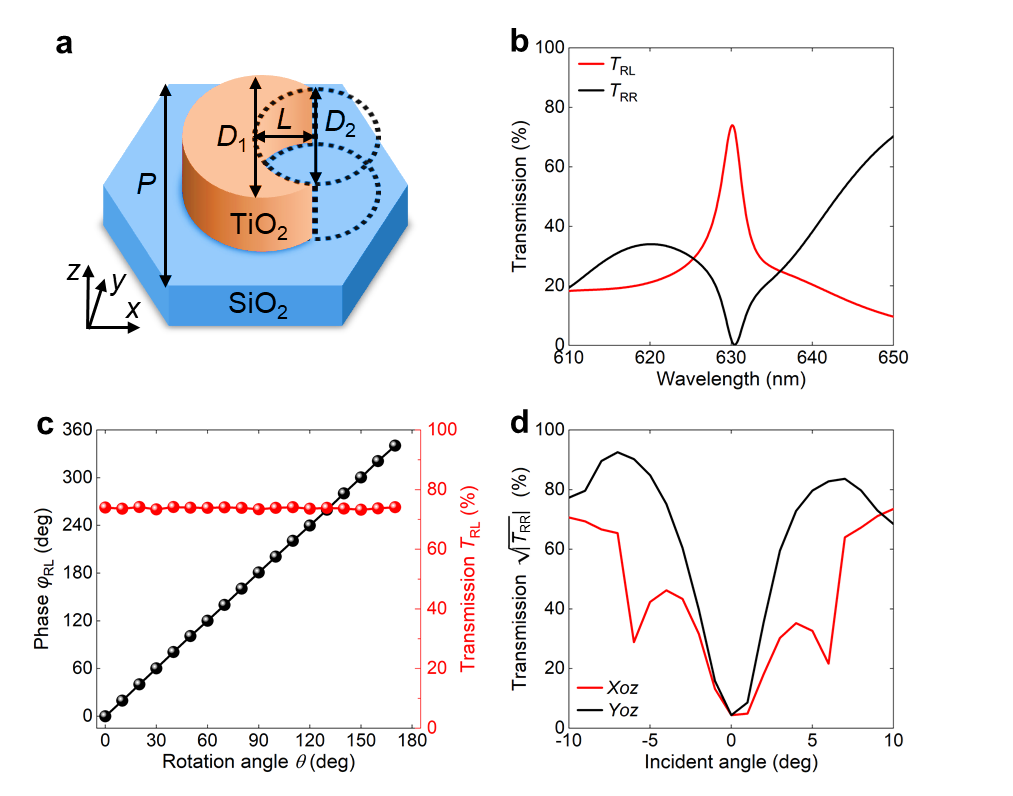


**Fig. S12: Meta-atom design in the visible. a**, Schematic diagram. Geometric parameters: *P* = 450 nm, *D*_1_ = 290 nm, *D*_2_ = 140 nm, *L* = 100 nm. **b**, Transmission spectra *T*_RL_ and *T*_RR_. **c**, Dependences of transmission *T*_RL_ and phase *φ*_RL_ on the rotation angle. **d**, Transmission coefficients in *xoz* and *yoz* planes as a function of incident angle.

To demonstrate the universality of the proposed functionality, we have designed the titanium dioxide meta-atom placed on the silica substrate for the visible band. Results are shown in Fig. S12. The resonant wavelength is 630 nm with a high quality factor of ~200. A polarization conversion efficiency of 74% is achieved, accompanied by a robust geometric phase. The unconverted component is minimal (~0) with an incident-angle dependence, enabling the transmission of high spatial frequency information. These effects are crucial for achieving high-quality-factor multiplexing imaging in the visible.

**Supplementary references**

1. Cotrufo M., Arora A., Singh S., Alù A. Dispersion engineered metasurfaces for broadband, high-NA, high-efficiency, dual-polarization analog image processing. *Nat. Commun.* **14**, 7078 (2023).

2. Malek S. C., Overvig A. C., Alu A., Yu N. Multifunctional resonant wavefront-shaping meta-optics based on multilayer and multi-perturbation nonlocal metasurfaces. *Light: Sci. Appl.* **11**, 246 (2022).

3. Lawrence M., Barton III D., Dixon J., Song J. H., van de Groep J., Brongersma M. L., Dionne J. A. High quality factor phase gradient metasurfaces. *Nat. Nanotechnol.* **15**, 956-961 (2020).

4. Lin L., Hu J., Dagli S., Dionne J. A., Lawrence M. Universal narrowband wavefront shaping with high quality factor meta-reflect-arrays. *Nano Lett.* **23**, 1355-1362 (2023).

5. Huo P., Zhang C., Zhu W., Liu M., Zhang S., Zhang S., Chen L., Lezec H. J., Agrawal A., Lu Y., Xu T. Photonic Spin-Multiplexing Metasurface for Switchable Spiral Phase Contrast Imaging. *Nano Lett.* **20**, 2791-2798 (2020).

6. Zhang Y., Lin P., Huo P., Liu M., Ren Y., Zhang S., Zhou Q., Wang Y., Lu Y.-q., Xu T. Dielectric Metasurface for Synchronously Spiral Phase Contrast and Bright-Field Imaging. *Nano Lett.* **23**, 2991-2997 (2023).

7. Badloe T., Kim Y., Kim J., Park H., Barulin A., Diep Y. N., Cho H., Kim W.-S., Kim Y.-K., Kim I., Rho J. Bright-Field and Edge-Enhanced Imaging Using an Electrically Tunable Dual-Mode Metalens. *ACS Nano* **17**, 14678-14685 (2023).
